# Feature Selection for Speech Emotion Recognition in Spanish and Basque: On the Use of Machine Learning to Improve Human-Computer Interaction

**DOI:** 10.1371/journal.pone.0108975

**Published:** 2014-10-03

**Authors:** Andoni Arruti, Idoia Cearreta, Aitor Álvarez, Elena Lazkano, Basilio Sierra

**Affiliations:** 1 Computer Science Faculty (University of the Basque Country), San Sebastián, Spain; 2 Vicomtech-IK4 Research Alliance, San Sebastián, Spain; University of Barcelona, Spain

## Abstract

Study of emotions in human–computer interaction is a growing research area. This paper shows an attempt to select the most significant features for emotion recognition in spoken Basque and Spanish Languages using different methods for feature selection. RekEmozio database was used as the experimental data set. Several Machine Learning paradigms were used for the emotion classification task. Experiments were executed in three phases, using different sets of features as classification variables in each phase. Moreover, feature subset selection was applied at each phase in order to seek for the most relevant feature subset. The three phases approach was selected to check the validity of the proposed approach. Achieved results show that an instance-based learning algorithm using feature subset selection techniques based on evolutionary algorithms is the best Machine Learning paradigm in automatic emotion recognition, with all different feature sets, obtaining a mean of 80,05% emotion recognition rate in Basque and a 74,82% in Spanish. In order to check the goodness of the proposed process, a greedy searching approach (FSS-Forward) has been applied and a comparison between them is provided. Based on achieved results, a set of most relevant non-speaker dependent features is proposed for both languages and new perspectives are suggested.

## Introduction

Affective computing, a discipline that develops devices for detecting and responding to user’s emotions [1], is a growing research area [2] in Human Computer Interaction (HCI). The main objective of affective computing is to capture and process affective information with the aim of enhancing and naturalizing the communication between the human and the computer. Within affective computing, affective mediation uses a computer-based system as intermediary in the communication of people, reflecting the emotion the interlocutors may have [1]. Affective mediation tries to minimize the filtering of affective information carried out by communication devices, because they are usually devoted to the transmission of verbal information and therefore, miss nonverbal information [3]. There are other applications in this type of mediated communication, for example, textual telecommunication (affective electronic mail, affective chats, etc.). Speech Emotion Recognition (SER) is also a very active research field in HCI [4]. Concerning to this topic, Ramakrishnan and El Emary [5] propose several types of applications to show the importance of techniques used in SER.

Affective databases are a good chance for developing affective applications, either for affective recognizers or either for affective synthesis. This paper presents a study aimed for giving a new step towards searching relevant speech features in automatic SER area for Spanish and Basque languages, using an affective database. This study is based on two previous works and its main objective is to analyse the results using the whole set of features which come from both of them. Moreover, it tries to extract the most relevant features related with the emotions in speech. Although all studies have started being speaker-dependent, in the extraction of relevant features the aim is to achieve a speaker-independent recognizer.

The three phases are the following: (a) using a group of 32 speech features [6]; (b) using a different group containing a total of 91 features [7]; and (c) finally, merging both groups, adding up a total of 123 different features.

Several Machine Learning (ML) techniques have been applied to evaluate their usefulness for SER. In this particular case, techniques based on evolutionary algorithms (EDA) have been used in all phases to select feature subsets that noticeably optimize the automatic emotion recognition success rate.

### Related work

Theories of emotions proposed by cognitive psychologists are a useful starting point for modelling human emotions. Although several theoretical emotional models exist, the most commonly used models of emotions are dimensional [8] and categorical [9], [10] ones. For practical reasons, categorical models of emotions have been more frequently used in affective computing. For example, in [11] several algorithms that recognize eight categories of emotions based on facial expressions are implemented. Oudeyer [12] has developed such algorithms for production and recognition of five emotions based on speech features. Authors such as Ekman and Friesen [13] suggest the *universality* of six basic categorical emotions and think that facial expressions for these six emotions are expressed and recognized in all cultures.

In [14], a study about the words that Basque-speaking people understand as emotions-related ones is presented and the hierarchical and family resemblance structure of the most prototypical 124 concepts that are represented as emotions are mapped. The hierarchical cluster analysis of collected data reveals two large superordinate categories (positive and negative) and five large basic level categories (love, happiness, anger, fear and sadness), which contain several subordinate level categories. They notice that those basic categories can also be found in similar studies made in Indonesia and United States of America.

Apart from models, there are also some studies related to expression and detection of emotions. In this way, Lang [8] proposed that three different systems would be implied in the expression of the emotions and that could serve like indicators to detect the emotion of the user:

- Verbal information: reports about perceived emotions described by users.
- Behavioural information: facial and postural expressions and speech paralinguistic features.
- Psychophysiological answers: such as heart rate, galvanic skin response -GSR-, and electroencephalographic response.

Verbal, behavioural and psychophysiological correlates of emotions should be taken into account when possible. Correlations among these three systems can help computers interpreting ambiguous emotions. For instance, a person with apraxia could have problems in the articulation of facial gestures, but subjective information written down with assistive technology can be used by a computer to interpret her/his emotional state. In that sense, more specific models or theories which describe the components of each system of expression can be found in the literature and selected according to the particular case, such as a dictionary of emotional speech [15], acoustic correlates of speech [10], sub-syllabic and pitch spectral features [16] or facial expressions [9].

On the other hand, affective resources, such as affective stimuli databases, provide a good opportunity for training affective applications, either for affective synthesis or for affective recognizers based on classification via Artificial Neural Networks, Hidden Markov Models, Genetic Algorithms (GAs), or similar techniques (see for example, [17] and [18]). These type of databases usually record information such as images, sounds, psychophysiological values, etc. There are some references in the literature that present affective databases and their characteristics. Cowie et al. [19] listed the major contemporary databases, emphasising those which are naturalistic or induced, multimodal, and influential. Other interesting reviews are the ones provided in [20] and [21].

Most of these references of affective databases are related to English, while other languages have less resources developed, especially the ones with relatively low number of speakers; this is the case of Basque Language. To our knowledge, the first affective database in Basque is the one presented by Navas et al. [22]. Concerning to Spanish, the work of Iriondo et al. [23] stands out; and relating to Mexican Spanish, the work of Caballero-Morales [24] can be highlighted.

RekEmozio database is a multimodal bilingual database for Spanish and Basque [25], which also stores information that came from processes of some global speech features extraction for each audio recording. Some of these features are prosodic features while others are quality features.

As in the case of affective databases, most emotional speech recognition systems are related to English. For languages such as Basque and Spanish much less emotional speech recognition systems have been developed. For Basque, the work of Luengo et al. [26] is noticeable. For Spanish, works such as [27] can be found in the literature. Another example is the work of Hozjan and Kačič [28], which studies multilingual emotion recognition and includes Spanish language. In this work, 26 high-level (AHL) features and 14 database-specific emotional (DSE) features were used. AHL are statistical presentations of low-level features (low-level features are composed from pitch, derivative of pitch, energy, derivative of energy, and duration of speech segments). DSE features are a set of speaker specific emotional features. Emotion recognition was performed using artificial neural networks and results were obtained using the *max-correct* evaluation method. Taking speaker-dependent emotion recognition into account, the average of *max-correct* with AHL features was 55.21% and for recognition with DSE features 45.76%. An aspect to consider is whether cultural and linguistic variations can modify emotional speech features. This aspect has been analysed in studies such as [29], [12] and [30]. In [29], an experimental study is performed comparing Spanish and Swedish cultures. However, it must be highlighted that no reference has been found in literature about Basque language being analysed in the context of cross-cultural studies related to speech. It must also be stated that few common speech features are provided in studies where Spanish language is present and that most cross-cultural studies found in literature are based on facial expression analysis.

ML paradigms take a principal role in some works related to SER found in the literature [31]. Some papers describe works performed using several classification methods. Support Vector Machines (SVM) and Decision Trees (DT) are compared to identify relevant emotional states from prosodic, disfluency and lexical cues extracted from the real-life spoken human-human interactions in [32]. Authors such as Pan et al. [33] also apply the SVM method to classify emotions in speech, using two emotional speech databases: Berlin German and Chinese. In [34], authors developed a hybrid system capable of using information from faces and voices to recognize people’s emotions. Three ML approaches are considered by Shami and Verhelst [35], K-nearest neighbours (KNN), SVM and Ada-boosted decision trees, applied to four emotional speech databases: Kismet, BabyEars, Danish, and Berlin. Rani et al. [36] presents a comparative study of four ML methods (KNN algorithm, Regression Trees (RT), Bayesian Networks and SVM) applied to the affect recognition domain using physiological signals. In [37] a system that recognizes human speech emotional states using a neural network classifier is proposed.

Different types of features (spectral, prosodic) for laughter detection were investigated by Truong and van Leeuwen [38] using different classification techniques (Gaussian Mixture Models, SVM, Multi Layer Perceptron). In [12] a large-scale data mining experiment about the automatic recognition of basic emotions in informal everyday short utterances is presented. A large set of ML algorithms is compared, ranging from Neural Networks, SVM or DT, together with 200 features, using a large database of several thousand examples, showing that the difference of performance among learning schemes can be substantial, and that some features which were previously unexplored are of crucial importance; several schemes are emerging as candidates for describing pervasive emotion.

It has to be pointed out the work by Schröder [39], which provides a wide list of references concerning emotional speech features. Most of these references are related to English and the features used by referenced authors are the most commonly found in the literature. In terms of emotional speech features for Basque, to authors’ knowledge, the work of Navas et al. [40] is the unique work and it also uses some of the most common features found. This situation is similar for Spanish, there are few references and some of most common features tend to be used [23], [41], [42]. On the other hand, in [43] and [44], a different approach of how to treat the signal that adds new and interesting features for the study of the emotions in the voice is presented.

Some works about feature selection for emotion recognition have been found in literature: in [45] Fast Correlation Based Filter is applied to select the attributes that take part in a Neural Network classifier; in [46], selection is performed by an expert; in [47] a non-linear dimensionality reduction is used to carry out the recognition process; Picard et al. [48] present and compare multiple algorithms for feature-based recognition of emotional state from this data; the work by Cowie et al. [19] is related with this paper in the sense that a Feature Selection method is used in order to apply a Neural Network to emotion recognition in spoken English, although both, the method chosen to perform the Feature Subset Selection (FSS) and the learning paradigms are different.

## Materials and Methods

As it is mentioned before, several ML techniques have been applied to evaluate their usefulness for SER and to obtain relevant emotional speech features. To fulfil this objective, a corpus has been used to extract several features. Next subsections describe this corpus and the ML paradigms used for classification purposes during the experimental phase.

### Corpus

There are few affective corpuses developed for Spanish language, and even less for Basque. The database used in this work has been Rekemozio, that contains instances of both languages and is the only alternative found for Basque. The creation and validation of this multimedia database, that includes video and audio recordings, is described in [21]. In our work, we only use the spoken material. Rekemozio uses a categorical model based on Ekman’s six basic emotions [13] (Sadness, Fear, Joy, Anger, Surprise and Disgust), and also considers a Neutral emotion category. In their work Ekman and Friesen suggested that they are universal for all cultures. Table 1 summarizes the scope of RekEmozio database, presenting its relevant features.

**Table 1:** Summary of RekEmozio database scope for recordings.

Language	#Actors	#male/#female	Mean Age (std dev)
Basque	7	4/3	31.3 (5.2)
Spanish	10	5/5	30.7 (4.1)
Overall	17	9/8	30.9 (4.4)

RekEmozio database recordings were carried out by skilled actors and actresses, and contextualized by means of audiovisual stimuli (154 audio stimuli and 6 video stimuli per actor). They were asked to read a set of words and sentences (both semantically and non-semantically relevant) trying to express emotional categories by means of voice intonation and facial expression. Regarding to spoken material, in Table 2, the amount of text used is pointed out, while Table 3 shows the length of the recordings (see [25] for more details).

**Table 2:** Amount of text used for both Spanish and Basque languages.

Text unit	Specific foreach emotion	Used in allemotions	Total	Per actor
Words	35	5	40	70
Sentences	21	3	24	42
Paragraphs	21	3	24	42
Total	77	11	88	154

**Table 3:** Lengths of RekEmozio database’s audio recordings.

Language	Recording’s lengths
Basque	130’41”
Spanish	166’17”
Total	296’58”

It should be noted that the database is validated [21]. It is considered that training affective recognizers with subject validated databases will enhance the effectiveness of recognition applications. Fifty-seven volunteers participated in the validation, and results of the categorical test allowed to conclude that the 78% of audio stimuli were valid to express the intended emotion as the recognition accuracy percentage was over 50%.

### Emotional feature extraction

One of the most important questions for automatic SER is which features should be extracted from the voice signal. Previous studies show that it is difficult to find specific voice features valid as reliable indicators of the emotion present in the speech [49].

Therefore, as a first step, an in-depth literature review of emotional speech features was carried out. After reviewing the state-of-the-art, in the first phase, a number of features which had been frequently used in other similar studies [40], [23], [41], where selected and checked. Using a 20 ms frame-based analysis, with an overlapping of 10 ms, information related to prosody, such as the fundamental frequency, energy, intensity and speaking rate, was extracted obtaining a total of 32 features. In this phase, encouraging results were obtained applying ML classification techniques.

In a second phase it was decided to study additional features that could provide information about the emotion expressed in the speech. Tato et al. [43] proposed new interesting formulas to extract information regarding emotions from speech, and also defined a novel technique for signal treatment, not only extracting information by frames, but by regions consisting of more than three consecutive frames, either for the analysis of voice and unvoiced parts. Before adding this information consisting of 91 new features to those used in the first phase, the effectiveness of these new features was tested using the same ML paradigms, to compare the results obtained in both phases.

After verifying the effectiveness of the classification procedures and the features selected in the first two phases, it was decided to compile all the features concerning emotional information in a third and final phase, obtaining a final set of 123 speech features as input for the previous ML paradigms.

All these features are divided as follows:

- Prosodic Features: model the F0, energy, voiced and unvoiced regions, pitch derivative curve and the relations between the features as is proposed in [50] and [44] (see Table 4).
- Spectral Features: formants and energy band distribution (see Table 5).
- Quality Features: related with the voice quality, such as harmonicity to noise ratio and active level in speech (see Table 6).

**Table 4:** Prosodic Features extracted for each validated recording.

Feature class	Description	Computed values
FundamentalFrequency	F0 curve in thevoiced parts.Estimation basedon Sun algorithm.	Maximum and its position, minimum and its position, mean, variance, standard deviation, maximum positive slope in contour, regression coefficient and its mean square error.
		Pitch derivative based features: maximum, minimum, mean, variance, regression coefficient and its mean square error.
Energy	Energy, RMSenergy andLoudness.	Maximum and its position, minimum and its position, mean, variance, regression coefficient and its mean square error.
		RMS: maximum, minimum, mean, range, variance and standard deviation.
		Loudness: absolute loudness based on Zwicker’s model.
Voiced/Unvoiced	Features basedon Voiced andUnvoiced framesand regions.	F0 value of the first and last voiced frames, number of voiced and unvoiced frames and regions, length of the longest voiced and unvoiced regions, ratio of number of voiced and unvoiced frames and regions.
Relations	Relations amongseveral features.	Mean, variance, mean of the maximum, variance of the maximum, mean of the pitch ranges and mean of the flatness of the pitch based on every voiced region pitch values.
		Pitch increasing and decreasing in voiced parts as well as the mean of the voiced regions duration.
		Many features related with the energy among the voiced regions, such as global energy mean, vehemence, mean of the flatness and tremor in addition to others.
Rhythm	Alternation betweenspeech and silence.	Duration of voice, silence, maximum voice, minimum voice, maximum silence and minimum silence in the whole utterance are computed.

**Table 5:** Spectral Features extracted for each validated recording.

Feature class	Description	Computed values
Formants	Resonance characteristicsof the vocal tract.	Mean of the first, second and third formant frequencies and their bandwidths among all voiced region as well as the mean, maximum and range of the second formant ratio.
Critical Bands	Energy in severalfrequency bands,using two differentspectral distributions.	Energy in three frequency bands: low band (0–1300 Hz), medium band (1300–2600 Hz) and high band (2600–4000 Hz).
		Energy in four frequency bands: (0 - F0 Hz), (0–1000 Hz), (2500–3500 Hz) and (4000–5000 Hz).
		Relative energy in each band for voiced parts of utterance.

**Table 6:** Quality Features extracted for each validated recording.

Feature class	Description	Computed values
Harmonicity to noise ratio	Ratio of the energy ofharmonic frames to theenergy of remainingpart of the signal.	Maximum harmonicity, minimum, mean, range and standard deviation.
Jitter	Pitch perturbationin vocal chordsvibration.	Cycle-to-cycle variation of pitch.
Shimmer	Energy perturbationin vocal chordsvibration.	Cycle-to-cycle variation of energy.
Active level	Signal activelevel features.	Maximum, minimum, mean and variance of the speech active level among the voiced regions.

### Machine Learning standard paradigms used

In the supervised learning task, the main goal is to construct a model or a classifier able to manage a classification task with an acceptable accuracy. With this aim, some variables are to be used in order to identify different elements, the so called predictor variables. In the present problem, each sample is composed by a set of speech related values, while the label value is one of the seven emotions identified.

We brieflyintroduce the single paradigms used in our experiments. These paradigms come from the ML family and are 4 well-known supervised classification algorithms. As seen before, the number of choices when selecting a classifier is very large, and in this work, being the main goal the feature selection for Speech Emotion Recognition, we have chosen to use simple paradigms, with long tradition in different classification tasks and with different approaches to learning.

#### Decision Trees

A *Decision Tree* consists of nodes and branches to partition a set of samples into a set of covering decision rules. In each node, a single test or decision is made to obtain a partition. The starting node is usually referred as the root node. In each node, the goal is selecting an attribute that makes the best partition between the classes of the samples in the training set [51] and [52]. In our experiments, two well-known decision tree induction algorithms are used, ID3 [53] and C4.5 [54].

#### Instance-Based Learning

Instance-Based Learning (IBL) has its root in the study of Nearest Neighbour algorithm [31] in the field of ML. The simplest form of Nearest Neighbour (NN) or KNN algorithms simply store the training instances and classify a new instance by predicting the same class its nearest stored instance has or the majority class of its k nearest stored instances have, respectively, according to some distance measure as described in [55]. The core of this non-parametric paradigm is the form of the similarity function that computes the distances from the new instance to the training instances, to find the nearest or k-nearest training instances to the new case. In our experiments the IB paradigm is used, an inducer developed in the MLC++ project [56] and based on the works of Aha et al. [57] and Wettschereck [58].

#### Naive Bayes classifiers

The Naive-Bayes (NB) rule [59] uses the Bayes theorem to predict the class for each case, assuming that the predictive genes are independent given the category. To classify a new sample characterized by *d* genes **X** = (*X1,X2,…,Xd*), the NB classifier applies the following rule:
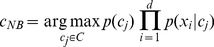
(1)where *c_NB_* denotes the class label predicted by the NB classifier and the possible classes of the problem are grouped in *C* = *{c_1_,…,c_n_}*. A normal distribution is assumed to estimate the class conditional densities for predictive genes. Despite its simplicity, the NB rule has obtained better results than more complex algorithms in many domains.

### Increasing the Accuracy by Feature Subset Selection

The goal of a supervised learning algorithm is to induce a classifier that allows us to classify new examples E* = e_n+1_,…,e_n+m_ that are only characterized by their *d* descriptive features. To generate this classifier we have a set of *n* samples *E = e_1_,…,e_n_*, characterized by *d* descriptive features *X = X_1_,…,X_d_* and the class label *C = w_1_,…,w_n_* to which they belong. ML can be seen as a data-driven process where, putting little emphasis on prior hypotheses a general rule is induced for classifying new examples using a learning algorithm. Many representations with different biases have been used to develop this classification rule. Here, the ML community has formulated the following question: “*Are all of these d descriptive features useful for learning the classification rule?*” Trying to respond to this question the FSS approach appears, which can be reformulated as follows: *given a set of candidate features, select the best subset under some learning algorithm*.

This dimensionality reduction made by a FSS process can carry out several advantages for a classification system in a specific task:

- Reduction in the cost of data acquisition
- Improvement of the comprehensibility of the final classification model
- Faster induction of the final classification model
- Improvement in classification accuracy

The attainment of higher classification accuracies is the usual objective of ML processes. It has been long proved that the classification accuracy of ML algorithms is not monotonic with respect to the addition of features. Irrelevant or redundant features, depending on the specific characteristics of the learning algorithm, may degrade the predictive accuracy of the classification model. In this work, FSS objective will be the maximization of the performance of the classification algorithm. In addition, with the reduction in the number of features, it is more likely that the final classifier is less complex and more understandable by humans.

Once the objective is fixed, FSS can be viewed as a search problem, with each state in the search space specifying a subset of the possible features of the task. Exhaustive evaluation of possible feature subsets is usually unfeasible in practice because of the large amount of computational effort required. Many search techniques have been proposed to solve FSS problem when there is no knowledge about the nature of the task, carrying out an intelligent search in the space of possible solutions. As randomized, evolutionary and population-based search algorithm, Genetic Algorithms (GAs) have long been used as the search engine in the FSS process. GAs need crossover and mutation operators to make the evolution possible.

#### Feature Subset Selection

As reported by Aha and Bankert [60], the objective of feature subset selection in ML is to *“reduce the number of features used to characterize a dataset so as to improve a learning algorithm’s performance on a given task”.* The objective will be the maximization of the classification accuracy in a specific task for a certain learning algorithm; as a collateral effect the number of features to induce the final classification model will be reduced. The feature selection task can be exposed as a search problem, each state in the search space identifying a subset of possible features. A partial ordering on this space, with each child having exactly one more feature than its parents, can be stated.

In order to state the FSS as a search problem, the following aspects must be identified:

- The starting point in the space. It determines the direction of the search. One might start with no features and successively add them, or one might start with all the features and successively remove them. One might also select an initial state somewhere in the middle of the search space.
- The organization of the search. It determines the strategy of the search in a space of size 2d, where d is the number of features in the problem. Roughly speaking, the search strategies can be optimal or heuristic. Two classic optimal search algorithms which exhaustively evaluate all possible subsets are depth-first and breadth-first [61]. Otherwise, Branch & Bound search [62] guarantees the detection of the optimal subset for monotonic evaluation functions without the systematic examination of all subsets.
- The evaluation function. It measures the effectiveness of a particular subset of features after the search algorithm has chosen it for examination. Being the objective of the search its maximization, the search algorithm utilizes the value returned by the evaluation function to help guide the search. Many measures carry out this objective regarding only the characteristics of the data, capturing the relevance of each feature or set of features to define the target concept. As reported by John et al. [63], when the goal of FSS is the maximization of the accuracy, the features selected should depend not only on the features and the target concept to be learned, but also on the learning algorithm.

Two factors can make difficult the implementation of FSS [64]: the number of features and the number of instances. One must bear in mind that the learning algorithm used in the searching scheme requires a training phase for every possible solution visited by the FSS search engine and this can be very time consuming.

One of the first approximations to FSS mentioned in the literature consists of performing a greedy (or Hill Climbing) search. Taking an empty as the initial variable set, the method attempts to include the variable that, at each step, maximizes the accuracy. The process stops when the inclusion of any variable does not show an improvement in the accuracy. This method is known as FSS-Forward.

More complex approximations for feature selection use genetic based operators as main searching engines.

#### Estimation of Distribution Algorithms as searching paradigm

Genetic Algorithms [65] are one of the best known techniques for solving optimization problems. Their use has reported promising results in many areas but there are still some problems where GAs fail. These problems, known as deceptive problems, have attracted the attention of many researchers and as a consequence there has been growing interest in adapting the GAs in order to overcome their weaknesses.

The GA is a population based search method. First, a set of individuals (or candidate solutions to our optimization problem) is generated (a population), then promising individuals are selected, and finally new individuals which will form the new. population are generated using crossover and mutation operators.

**Figure 1:**
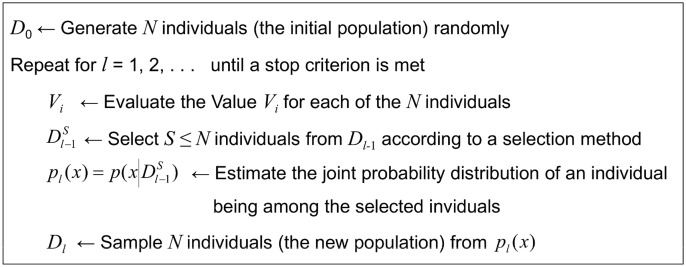
Main scheme of the Estimation of Distribution Algorithms (EDA) approach.

An interesting adaptation of this is the Estimation of Distribution Algorithm (EDA) [66] (see Figure 1). In EDA, there are neither crossover nor mutation operators, the new population is sampled from a probability distribution which is estimated from the selected individuals.

In this way, a randomized, evolutionary, population-based search can be performed using probabilistic information to guide the search. It is shown that although EDA approach process solutions in a different way to GAs, it has been empirically proven that the results of both approaches can be very similar [67]. In this way, both approaches do the same except that EDA replaces genetic crossover and mutation operators by means of the following two steps:

- A probabilistic model of selected promising solutions is induced,
- New solutions are generated according to the induced model.

The main problem of EDA resides on how the probability distribution *p_l_(x)* is estimated. Obviously, the computation of 2*n* probabilities (for a domain with *n* binary variables) is impractical. This has led to several approximations where the probability distribution is assumed to factorize according to a probability model (see [67] or [68] for a review).

The simplest way to estimate the distribution of good solutions assumes the independence between the features of the domain. New candidate solutions are sampled by only regarding the proportions of the values of all features independently to the remaining solutions. Population Based Incremental Learning (PBIL) [69], Compact Genetic Algorithm (cGA) [70] and Univariate Marginal Distribution Algorithm (UMDA) [71] are three algorithms of this type. They have worked well under artificial tasks with no significant interactions among features and so, the need for covering higher order interactions among the variables is seen for more complex or real tasks.

## Results and Discussion

**Figure 2:**
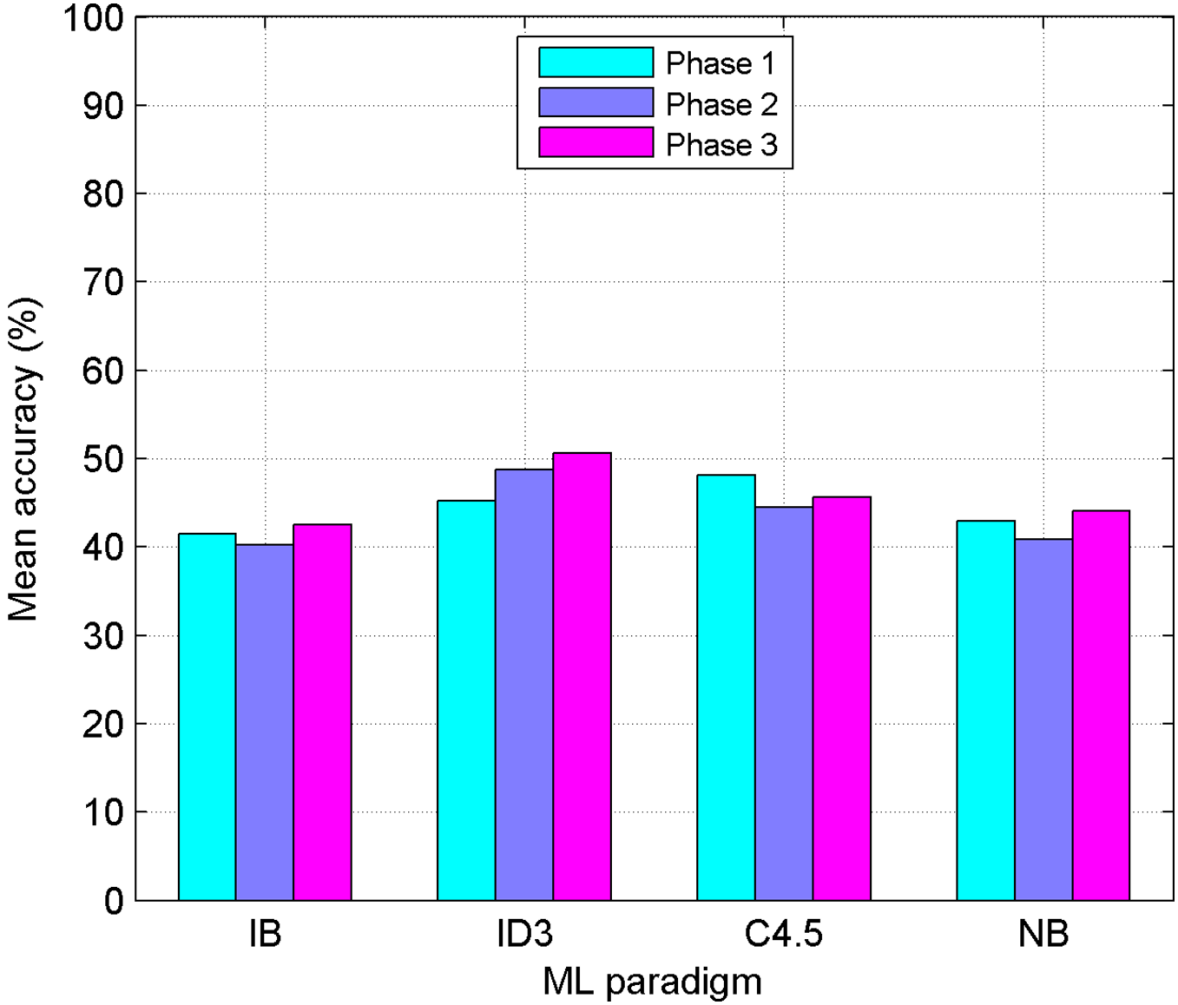
Results for the Basque Language without Feature Subset Selection. Performance comparison between four Machine Learning paradigms (IB: Instance Based, ID3: Decision Tree, C4.5: Decision Tree, NB: Naive-Bayes) without any kind of FSS. Mean accuracy obtained in the three phases, for the Basque language, is shown.

**Figure 3:**
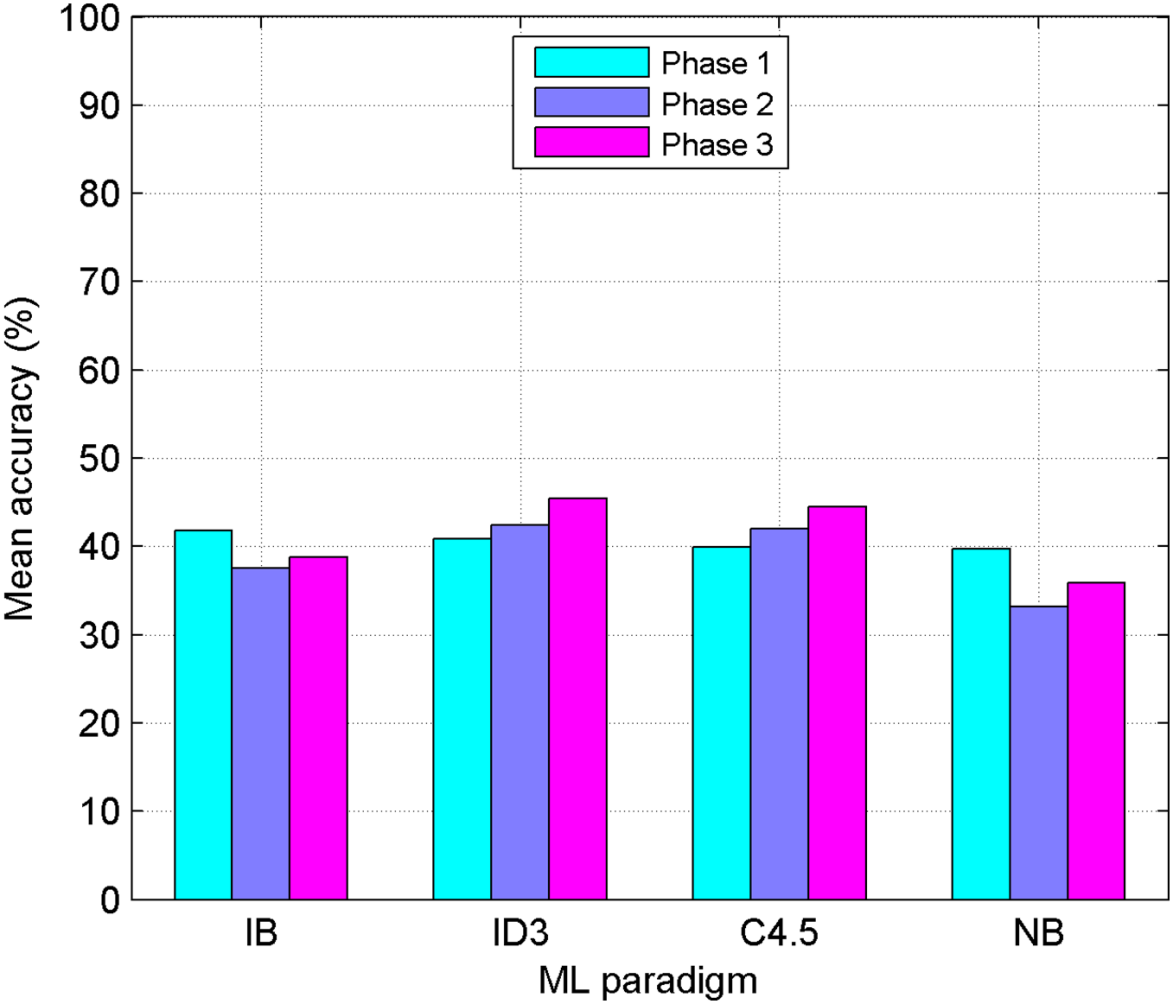
Results for the Spanish Language without Feature Subset Selection. Performance comparison between four Machine Learning paradigms (IB: Instance Based, ID3: Decision Tree, C4.5: Decision Tree, NB: Naive-Bayes) without any kind of FSS. Mean accuracy obtained in the three phases, for the Spanish language, is shown.

**Figure 4:**
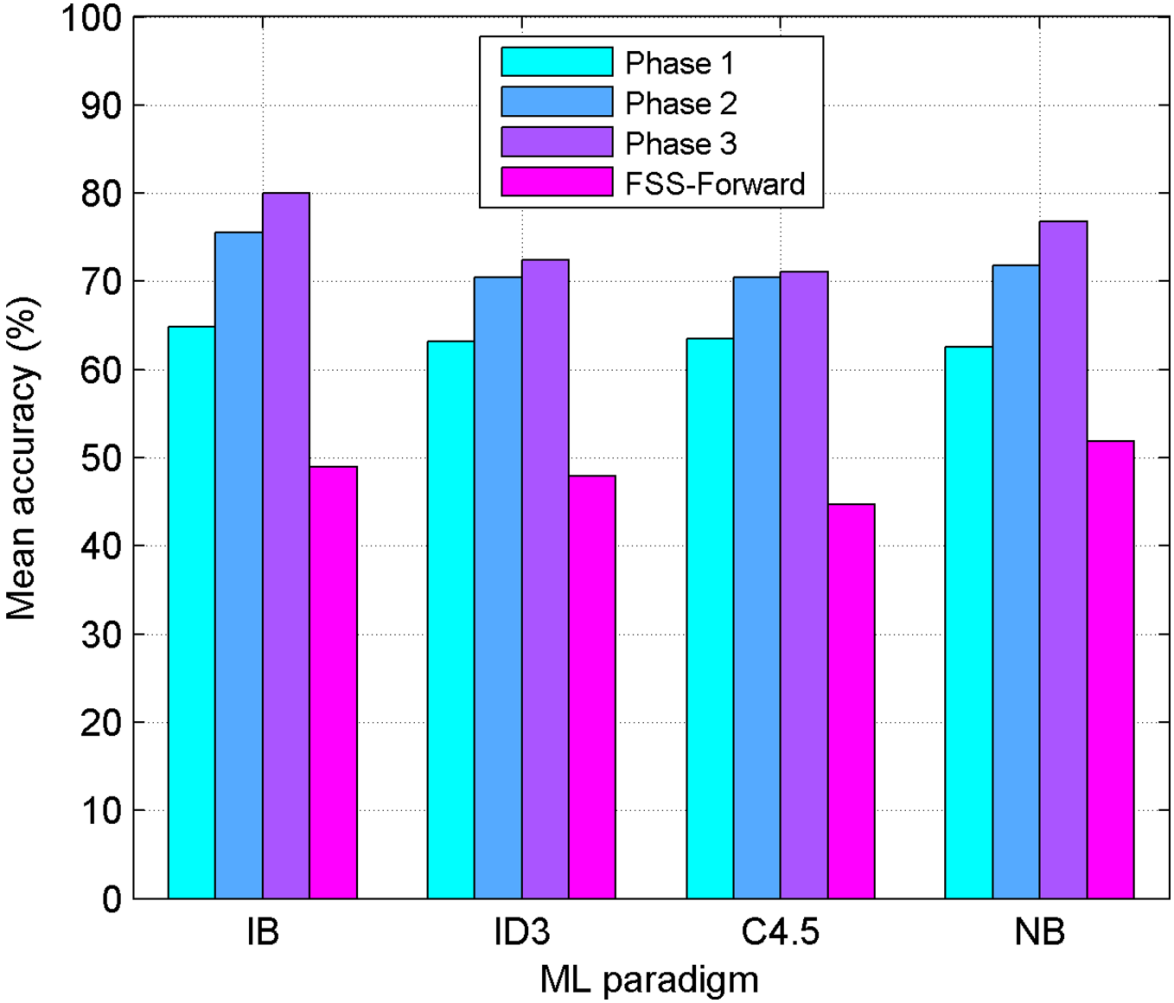
Results for the Basque Language using EDA Feature Subset Selection. Performance comparison between four Machine Learning paradigms (IB: Instance Based, ID3: Decision Tree, C4.5: Decision Tree, NB: Naive-Bayes) using EDA-FSS. Mean accuracy obtained in the three phases, for the Basque language, is shown. Results obtained with a standard FSS-Forward approach are also shown.

**Figure 5:**
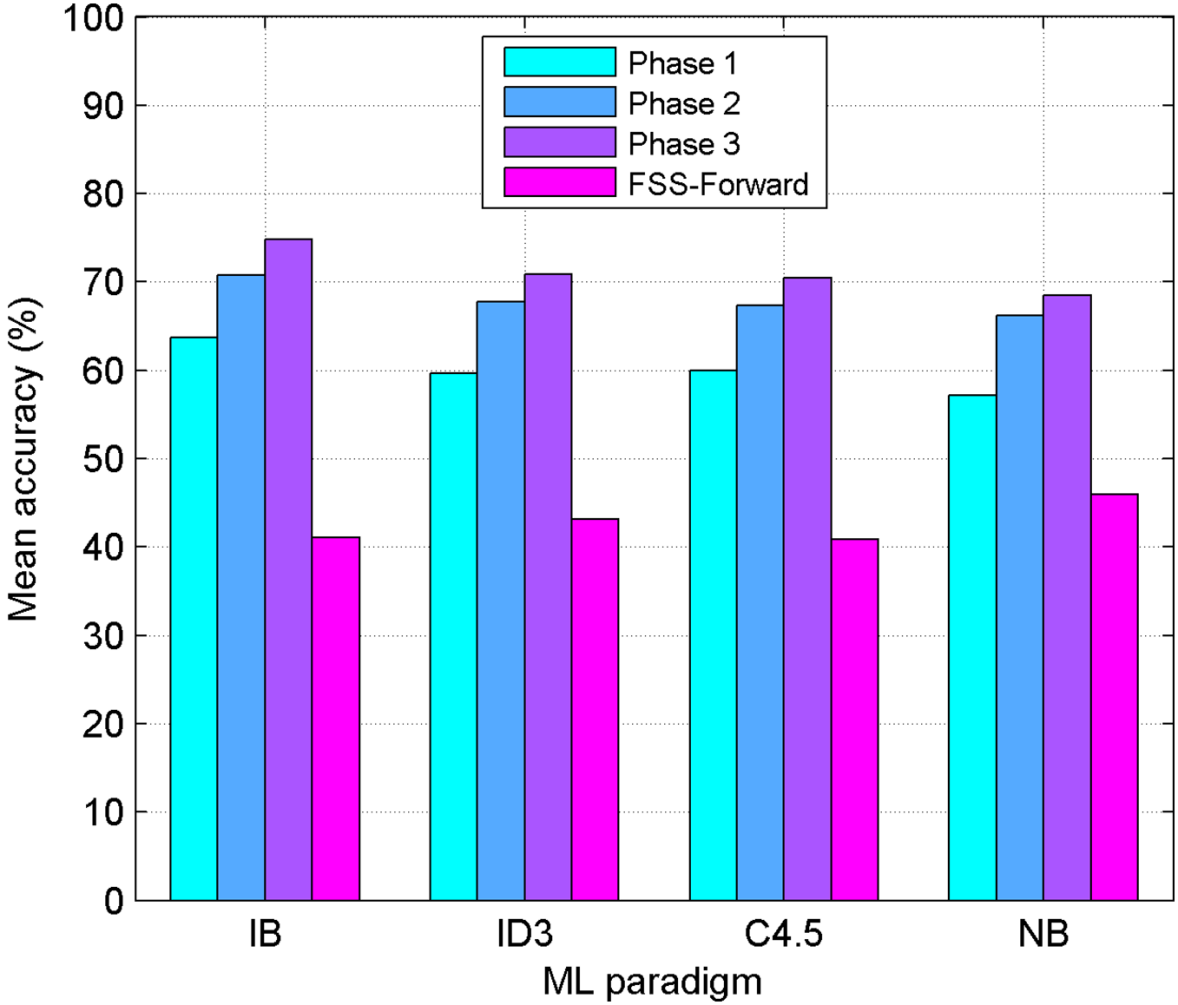
Results for the Spanish Language using EDA Feature Subset Selection. Performance comparison between four Machine Learning paradigms (IB: Instance Based, ID3: Decision Tree, C4.5: Decision Tree, NB: Naive-Bayes) using EDA-FSS. Mean accuracy obtained in the three phases, for the Basque language, is shown. Results obtained with a standard FSS-Forward approach are also shown.

The abovementioned methods have been applied over the crossvalidated datasets using the MLC++ library [56]. Each dataset corresponds to a single actor. As previously mentioned, experiments were carried out within three different phases. At first the initial 32 features have been employed; then, the second set of 91 new features has been used; finally, both sets have been joined completing a global set of 123 features. The datasets corresponding to the 17 actors can be found in Files S1–S17, each of them containing a feature matrix with 123 columns. Tables 7 to 18 show the results obtained for the three phases, applying the ML classifiers mentioned in previous section with and without FSS. Each column in these Tables represents a female (Fi) or male (Mi) actor, and mean values corresponding to each classifier/gender are also included. Last column presents the total average for each classifier in each language. Confusion Matrices corresponding to the best results obtained for each gender and language are also shown in Tables 19 to 22. In order to check the validity of proposed process, a greedy searching approach (FSS-Forward) has been applied. Tables 23 and 24 show the results obtained applying this method. A comparison among different phases and ML paradigms used is also provided (Figures 2 to 5). Finally, some statistical tests have been applied to check the significance of the results obtained in the third phase (Tables 25 and 26).

**Table 7:** 10-fold crossvalidation accuracy of first phase for actors in Basque.

	Female				Male					Total
	F1	F2	F3	mean	M1	M2	M3	M4	mean	
IB	35.38	48.79	35.23	39.80	44.17	49.32	36.89	40.91	42.82	41.52
ID3	38.71	45.45	44.70	**42.95**	46.67	46.97	43.26	51.14	47.01	45.27
C4.5	41.52	52.20	35.00	42.90	60.38	53.26	45.08	49.47	**52.04**	**48.13**
NB	42.95	45.76	37.65	42.12	52.20	44.09	36.21	41.44	43.48	42.90

**Table 8:** 10-fold crossvalidation accuracy of first phase for actors in Spanish.

	Female						Male						Total
	F1	F2	F3	F4	F5	mean	M1	M2	M3	M4	M5	mean	
IB	34.55	43.64	54.55	54.55	38.18	45.09	25.45	33.64	51.82	47.65	33.64	**38.44**	**41.77**
ID3	36.36	52.73	49.09	47.27	42.73	**45.63**	20.91	30.91	40.91	47.27	40.00	36.00	40.82
C4.5	30.91	50.00	46.36	43.64	42.73	42.72	29.09	31.82	46.36	42.73	35.45	37.09	39.91
NB	38.18	42.73	49.09	40.00	42.73	42.54	24.55	30.91	49.09	45.45	34.55	36.91	39.73

**Table 9:** 10-fold crossvalidation accuracy of first phase for actors in Basque applying EDA-FSS.

	Female				Male					Total
	F1	F2	F3	mean	M1	M2	M3	M4	mean	
IB	63.03	68.03	59.32	**63.46**	72.65	67.35	60.98	62.80	**65.94**	**64.88**
ID3	62.73	60.48	65.45	62.88	72.65	61.97	56.52	62.65	63.44	63.20
C4.5	60.23	65.98	60.00	62.07	71.82	62.80	60.08	63.56	64.56	63.49
NB	64.47	64.55	48.94	59.32	74.55	62.50	62.73	60.00	64.94	62.53

**Table 10:** 10-fold crossvalidation accuracy of first phase for actors in Spanish applying EDA-FSS.

	Female						Male						Total
	F1	F2	F3	F4	F5	mean	M1	M2	M3	M4	M5	mean	
IB	61.82	66.36	75.45	71.82	68.18	**68.72**	42.73	57.27	69.09	63.64	60.91	**58.72**	**63.72**
ID3	59.09	66.36	66.36	60.00	61.81	62.72	42.73	51.82	66.36	61.82	60.00	56.54	59.63
C4.5	57.27	62.73	64.55	65.45	63.64	62.72	43.64	56.36	65.45	64.55	56.36	57.27	60.00
NB	54.55	59.09	68.18	65.45	60.00	61.45	40.91	48.18	64.55	59.09	51.82	52.91	57.18

**Table 11:** 10-fold crossvalidation accuracy of second phase for actors in Basque.

	Female				Male					Total
	F1	F2	F3	mean	M1	M2	M3	M4	mean	
IB	34.00	42.91	33.91	36.94	56.18	41.00	36.91	36.82	42.73	40.25
ID3	49.45	45.91	46.78	**47.38**	54.27	44.00	51.45	49.45	**49.79**	**48.75**
C4.5	42.73	40.09	42.73	41.85	60.36	39.55	48.45	37.82	46.55	44.54
NB	39.82	31.00	46.45	39.09	60.36	29.91	36.91	41.44	42.16	40.84

**Table 12:** 10-fold crossvalidation accuracy of second phase for actors in Spanish.

	Female						Male						Total
	F1	F2	F3	F4	F5	mean	M1	M2	M3	M4	M5	mean	
IB	36.46	41.92	41.92	43.64	33.64	39.52	30.00	36.46	44.55	36.46	30.00	35.49	37.51
ID3	38.18	47.27	55.45	43.64	44.55	45.82	24.55	40.00	50.00	46.36	34.55	**39.09**	**42.46**
C4.5	42.73	48.18	50.91	50.91	45.45	**47.64**	21.82	39.09	46.36	48.18	27.27	36.54	42.00
NB	34.55	34.45	40.91	32.73	31.82	34.89	20.91	39.09	40.00	35.45	21.82	31.45	33.17

**Table 13:** 10-fold crossvalidation accuracy of second phase for actors in Basque applying EDA-FSS.

	Female				Male					Total
	F1	F2	F3	mean	M1	M2	M3	M4	mean	
IB	72.55	79.73	62.27	**71.52**	91.36	73.00	77.82	71.82	**78.50**	**75.50**
ID3	71.00	71.73	66.64	69.79	78.73	65.82	72.64	66.91	71.03	70.50
C4.5	67.73	75.91	68.09	70.58	76.73	65.82	69.91	68.91	70.34	70.44
NB	73.00	77.73	63.36	71.36	89.45	67.27	66.18	65.36	72.07	71.76

**Table 14:** 10-fold crossvalidation accuracy of second phase for actors in Spanish applying EDA-FSS.

	Female						Male						Total
	F1	F2	F3	F4	F5	mean	M1	M2	M3	M4	M5	mean	
IB	72.73	72.73	80.91	76.36	64.55	**73.46**	58.18	72.73	76.36	70.00	62.73	**68.00**	**70.73**
ID3	67.27	75.45	73.64	72.73	68.18	71.45	51.82	63.64	76.36	69.09	59.09	64.00	67.72
C4.5	70.91	75.45	74.55	64.55	66.36	70.36	54.55	63.64	80.91	66.36	56.36	64.36	67.35
NB	75.45	73.64	68.18	67.27	64.55	69.82	50.00	60.00	76.36	68.18	58.18	62.54	66.18

**Table 15:** 10-fold crossvalidation accuracy of third phase for actors in Basque.

	Female				Male					Total
	F1	F2	F3	mean	M1	M2	M3	M4	mean	
IB	36.00	46.82	33.82	38.88	59.45	44.36	40.45	36.55	45.20	42.49
ID3	49.55	47.64	39.91	**45.70**	61.00	49.27	53.36	50.36	**54.25**	**50.59**
C4.5	50.73	47.36	35.82	44.64	63.91	35.09	48.18	38.64	46.46	45.68
NB	43.73	40.91	40.91	41.85	58.36	37.09	46.64	40.82	45.73	44.07

**Table 16:** 10-fold crossvalidation accuracy of third phase for actors in Spanish.

	Female						Male						Total
	F1	F2	F3	F4	F5	mean	M1	M2	M3	M4	M5	mean	
IB	32.73	36.36	48.18	45.45	40.00	40.54	28.18	40.91	47.27	37.27	31.82	37.09	38.82
ID3	35.45	50.00	55.45	41.92	50.91	46.75	30.00	49.09	55.45	47.27	39.09	**44.18**	**45.47**
C4.5	44.55	51.82	57.27	49.09	45.45	**49.64**	25.45	44.55	46.36	45.45	34.55	39.27	44.46
NB	30.91	38.18	44.55	32.73	40.91	37.46	20.91	37.27	46.36	40.91	26.36	34.36	35.91

**Table 17:** 10-fold crossvalidation accuracy of third phase for actors in Basque applying EDA-FSS.

	Female				Male					Total
	F1	F2	F3	mean	M1	M2	M3	M4	mean	
IB	75.36	82.55	73.73	**77.21**	90.45	84.27	76.27	77.73	**82.18**	**80.05**
ID3	68.09	75.64	71.64	71.79	78.82	69.55	73.73	69.73	72.96	72.46
C4.5	69.82	77.73	68.09	71.88	78.64	64.91	66.91	71.45	70.48	71.04
NB	74.82	82.55	67.27	74.88	91.27	78.73	67.91	74.73	78.16	76.75

**Table 18:** 10-fold crossvalidation accuracy of third phase for actors in Spanish applying EDA-FSS.

	Female						Male						Total
	F1	F2	F3	F4	F5	mean	M1	M2	M3	M4	M5	mean	
IB	71.82	77.27	80.91	80.91	78.18	**77.82**	59.09	73.64	80.91	74.55	69.09	**71.42**	**74.82**
ID3	68.18	75.45	80.00	70.00	75.45	73.82	50.00	70.00	80.00	72.73	67.27	68.00	70.91
C4.5	67.27	73.64	80.00	71.82	70.91	72.73	52.73	70.00	76.36	75.45	66.36	68.18	70.46
NB	70.00	77.27	78.18	77.27	62.73	73.09	51.82	63.64	74.55	69.09	60.00	63.82	68.46

**Table 19:** Confusion Matrix of the *F2* Basque actor.

	Sadness	Fear	Joy	Anger	Surprise	Disgust	Neutral
Sadness	**20**	0	0	0	0	0	0
Fear	0	**6**	1	0	0	0	1
Joy	0	0	**14**	1	0	0	0
Anger	0	0	2	**14**	0	0	1
Surprise	1	1	1	0	**5**	1	1
Disgust	2	0	2	0	0	**5**	0
Neutral	1	0	0	0	0	0	**21**

**Table 20:** Confusion Matrix of the *M1* Basque actor.

	Sadness	Fear	Joy	Anger	Surprise	Disgust	Neutral
Sadness	**18**	0	0	0	0	0	2
Fear	1	**7**	0	0	0	0	0
Joy	0	0	**16**	1	0	0	0
Anger	0	0	2	**13**	0	1	1
Surprise	0	0	0	2	**8**	0	0
Disgust	0	0	0	0	0	**9**	0
Neutral	0	0	0	0	0	0	**22**

**Table 21:** Confusion Matrix of the *F3* Spanish actor.

	Sadness	Fear	Joy	Anger	Surprise	Disgust	Neutral
Sadness	**17**	0	0	0	0	0	1
Fear	0	**7**	2	0	1	1	0
Joy	0	0	**14**	2	2	0	2
Anger	0	0	1	**16**	0	0	0
Surprise	0	0	2	1	**11**	1	0
Disgust	1	0	1	0	0	**4**	3
Neutral	0	0	0	0	0	0	**20**

**Table 22:** Confusion Matrix of the *M3* Spanish actor.

	Sadness	Fear	Joy	Anger	Surprise	Disgust	Neutral
Sadness	**17**	0	0	0	0	0	1
Fear	0	**7**	2	1	0	1	0
Joy	0	1	**18**	0	0	0	1
Anger	0	0	1	**14**	0	2	0
Surprise	0	1	0	1	**10**	2	1
Disgust	0	0	3	1	2	**3**	0
Neutral	0	0	0	0	0	0	**20**

**Table 23:** 10-fold crossvalidation accuracy for Basque applying FSS-FORWARD to the whole set.

	Female				Male					Total
	F1	F2	F3	mean	M1	M2	M3	M4	mean	
IB	38.91	46.55	44.00	43.15	66.18	51.45	47.45	48.55	53.41	49.01
ID3	42.73	43.55	52.45	46.24	59.27	42.82	49.36	45.45	49.23	47.95
C4.5	47.18	49.45	36.00	44.21	63.00	33.73	39.64	43.64	45.00	44.66
NB	47.45	62.09	31.09	**46.88**	69.73	56.18	46.45	50.36	**55.68**	**51.91**

**Table 24:** 10-fold crossvalidation accuracy for Spanish using FSS-FORWARD to the whole set.

	Female						Male						Total
	F1	F2	F3	F4	F5	mean	M1	M2	M3	M4	M5	mean	
IB	45.45	46.36	56.36	52.73	32.73	46.73	23.64	26.36	47.27	44.55	35.45	35.45	41.09
ID3	38.18	45.45	60.00	48.18	45.45	47.45	26.36	40.91	44.55	42.73	40.00	38.91	43.18
C4.5	35.45	46.36	57.27	55.45	39.09	46.72	29.09	28.18	44.55	35.45	37.27	34.91	40.82
NB	45.45	54.55	53.64	61.72	40.91	**51.25**	28.18	38.18	53.64	49.09	34.55	**40.73**	**45.99**

**Table 25:** p-values obtained with Wilcoxon test comparing FSS methods.

Classifier	FSS-FWD > without FSS ?	EDA > FSS-FWD ?
All	**0,03667**	**3,89e-13**
IB	**0,02323**	**0,00016**
ID3	0,95359	**0,00016**
C4.5	0,92620	**0,00016**
NB	**0,00174**	**0,00016**

**Table 26:** p-values obtained with Wilcoxon test comparing the best classifier for each FSS method with the others classifiers.

FSS method	Classifier	> IB ?	> ID3 ?	> C4.5 ?	> NB ?
None	ID3	**0,00038**	1	0,06477	**0,00019**
Forward	NB	**0,00930**	0,05119	**0,00260**	1
EDA	IB	1	**0,00016**	**0,00019**	**9,155e-05**

### First phase

Tables 7 and 8 show the results obtained for the first phase, without FSS for Basque and Spanish languages respectively, while Tables 9 and 10 show the improvement obtained by selecting relevant features. Here, IB paradigm with FSS outperforms both Basque and Spanish results, improving previous ones in 16.75% and 21.95% respectively.

### Second phase

Results obtained using the second set of 91 features are reflected in Tables 11 and 12 (without FSS) and in Tables 13 and 14 (with FSS). ID3 is the best classifier for both languages when no FSS is applied. The results are slightly better than those obtained without FSS for the first phase, although the difference is not very significant. On the contrary, when FSS is applied to these second set of features the emotion classification performance is highly increased. Again, IB classifier stands out with an accuracy of 75.5% and 70.73% for Basque and Spanish, respectively. Compared to previous phase, accuracy is increased in a 10.62% for Basque and a 7.01% for Spanish.

### Third phase

In this experiment, a set of 123 predictor features is used. Here, ID3 results show a small increase of performance without FSS (1.84% Basque and 3.01% Spanish), but improvement obtained after applying FSS to this whole set is more impressive. The classification accuracy is 4.55% higher for Basque and 4.09% higher for Spanish compared to previous phase, rising the overall performance up to 80.05% (Basque) and 74.82% (Spanish) (see Tables 15 to 18).

Tables 19 to 22 show the Confusion Matrices corresponding to the best results obtained for each gender and language. As it could be seen, very few errors are found in the classification process after FSS is performed.

### FSS-Forward

To show the EDA searching process goodness, a greedy FSS searching approach (FSS-Forward) has also been applied. This method has only been tested for the third phase feature set, as it is only presented for comparison purposes. Obtained results are shown in Tables 23 and 24. The best results seem to be obtained with NB classifier for both languages, but classification performances are disappointing, as far as they are similar to those obtained using the initial set of 32 features without FSS.

### Results comparison among different phases

The bar diagram in Figure 2 compares the performance of the four ML paradigms used (IB, ID3, C4.5, NB) without any kind of FSS, for the Basque language. Same comparison is shown for the Spanish language in Figure 3. It can be seen how ID3 outstands for both languages; results obtained using the full set are 50.53% for Basque and 45.47% for Spanish.

Figures 4 and 5 make the same comparison (Basque and Spanish, respectively) but this time, the improvements obtained after applying FSS to the different feature subsets are shown. The first three bars in each classifier column correspond to EDA-FSS, while the fourth one represents the FSS-Forward approach. Here, IB outperforms the rest of the classifiers for both languages and best results are obtained when EDA-FSS is applied to the whole set of features.

It is worth emphasizing that the difference between the classification accuracies obtained with the initial set of 32 features without FSS and those obtained with the whole set of 123 features after applying FSS sum up a notable increase in average of 30.62% for the Basque language and 30.61% for the Spanish language.

### Statistical tests

As seen in previous subsections, EDA based FSS clearly improves classification accuracies for all subjects, in both languages, and with all the classifiers, but to extract other interesting conclusions about the goodness of classifiers and FSS-Forward procedure, the mean values for all subjects are not sufficiently significant, and some type of statistical test should be made.

We have used Wilcoxon signed-rank test [72], that is a non-parametric paired difference test, used to assess whether two population mean ranks differ. Specifically, we have used the right-sided version, which tests a hypothesis of the form X>Y?

Tables 25 and 26 show the p-values obtained by applying the test to various hypotheses. Only third phase feature set has been used for tests, and in all cases the sample to test is constructed using the classification accuracies obtained for all subjects (17 without distinguishing languages), for a given classifier and FSS strategy. In some cases we have put together the four types of classifiers, working with samples of 61 values.

A p-value is a nonnegative scalar from 0 to 1 that represents the probability of observing, under the null hypothesis, data as or more extreme than the obtained values. If the p-value is less than a certain significance level we say that the hypothesis is significantly valid. In tables 25 and 26, significant values (<5%) are in bold.

In Table 25 the improvement obtained with the different FSS strategies are compared. The second column shows that if we do not distinguish between classifiers, FSS-forward is significantly better than not using FSS, but the p-value is just down 5%. In fact, its behaviour depends strongly on the classifier, obtaining the best results for NB, but not improving significantly with ID3 and C4.5. The third column shows, as we already knew, that EDA-FSS significantly improves the results of FSS-forward in all cases.

In Table 26, the classifier with best results for each FSS methods is compared with the others. Without FSS, ID3 is significantly better than IB and NB. When features are selected with greedy FSS-forward method, NB is significantly better than IB and C4.5. Finally, when EDA-FSS is applied, IB clearly outperforms all the other classifiers.

### Most relevant features

The procedure employed to extract the most relevant features is based on the results and the features used in the third phase, where the best classification rates have been obtained and the whole set of features have been employed.

EDA based FSS has been applied for each of previous described ML paradigms, so each classifier has found its own relevant features for each actor. In order to identify the most relevant speech features for SER this estimation has been based on the paradigm which obtains the higher classification rate after applying FSS. As mentioned before, the classifier with the best results in most of the cases is the IB paradigm, except in a case of a male actor (M1) for Basque language (see Table 17). As overall IB can be considered the most adequate option for the defined task, IB paradigm resulting features have been taken into account to select the most relevant features, which have been extracted separately for Spanish and Basque languages on one hand and for gender on the other (see Tables 27 and 28).

**Table 27:** The most relevant features using the IB paradigm with EDA for Basque.

Feature class	Female	Male
FundamentalFrequency	Position of themaximum, minimum andits position, mean, varianceand mean square error ofthe regression coefficient.	Mean, variance, maximum positive slope in contour, mean square error of the regression coefficient.
		Mean of the derivative and mean square error of the regression coefficient of the derivative.
Energy	Maximum, mean, varianceand regression coefficient.	Maximum, minimum, mean, variance, mean square error of the regression coefficient.
	RMS maximum and mean.	RMS maximum and mean.
	Loudness.	Loudness
Voiced/Unvoiced	F0 value of the first andlast voiced frames andlength of the longestunvoiced region.	Ratio of number of voiced and unvoiced frames and number of frames.
Relations	Mean of the pitch meansin every regions andduration from beginningto pitch maximum.	Mean of the pitch means in every regions.
	Ratio of the energy maximum.	
Formants	Mean of the second andthird formant frequency,the bandwidths of the firstand second formants andmean of the second formant ratio.	Mean of the first, second and third formant frequency and the bandwidths of the first and second formants
Critical Bands	Energy in bands(0–1300 Hz),(0 - F0 Hz) and(2500–3500 Hz).	Energy in band (1300–2600 Hz). Energy in band (2500–3500 Hz) of whole the utterance divided by the energy over all frequencies
	Rate of the energy of thelongest region and energyover all the utterance.	Rate of energy in longest region and energy over all the utterance.
Harmonicity tonoise ratio	Range.	Range.
Jitter	Cycle-to-cyclevariation of pitch.	
Shimmer	Cycle-to-cyclevariation of energy.	
Active level	Maximum and mean.	Maximum and mean.

**Table 28:** The most relevant features using the IB paradigm with EDA for Spanish.

Feature class	Female	Male
Fundamental Frequency	Minimum, mean, varianceand regression coefficientand its mean square error.	Maximum, minimum, mean, variance and mean square error of the regression coefficient.
	Maximum, mean and meansquare error of theregression coefficientof the derivative	Mean of the derivative.
Energy	Maximum, minimum,mean, variance andregression coefficientand its mean square error.	Maximum and mean.
	RMS maximum,minimum and mean.	RMS value, maximum, mean.
	Loudness.	Loudness.
Voiced/Unvoiced	F0 value of the first andlast voiced frames andlength of the longest unvoicedregion, ratio of numberof voiced frames andnumber of frames.	F0 value of the first voiced frame, number of unvoiced frames, length of the longest unvoiced region, ratio of unvoiced regions.
Relations	Mean and variance of the pitch means in every regions.	Mean, variance, variance of the maximum, mean of the pitch ranges and mean of the flatness of the pitch based on every voiced region pitch values.
		Global energy mean among voiced regions
Rhythm	Duration of silence andmaximum voiced parts.	Duration of silence parts.
Formants	Mean of the first, secondand third formant frequencyand the bandwidths of thesecond and third formants.	Mean of the first formant frequency and the bandwidths of the first, second and third formants.
Critical Bands	Energy in bands (0–1300 Hz)and (2600–4000 Hz).Energy in bands (0–1000 Hz),(2500–3500 Hz)and of whole theutterance divided bythe energy over allfrequencies.	Energy in bands (0–1300 Hz) and (2600–4000 Hz). Energy in band (4000–5000 Hz) of whole the utterance divided by the energy over all frequencies.
	Rate of the energyof the longestregion and energyover all the utterance.	Rate of the energy of the longest region and energy over all the utterance.
Harmonicity tonoise ratio		Minimum
Shimmer		Perturbation cycle to cycle of the energy.
Active level	Maximum, minimum,mean and variance.	Maximum, mean and variance.

This information concerns to the features that EDA evolutionary algorithm selects more frequently for each actor. Given that the classification is speaker dependent, each actor may have different relevant features for each ML paradigm. These relevant features have been analyzed grouping actors by language and gender aiming at a partial independence of the actor. The purpose of this grouping is to shed more light on the impact that gender and language can have in the final features of each subgroup. The criterion to consider relevant a feature in a subgroup is that more than the 50% of the actors have that feature selected by the algorithm.

It must be highlighted that several features are common for all the categories, both for Spanish and Basque languages and for male and female gender, principally the prosodic features related with the Fundamental Frequency - the mean, variance, the mean square error of the regression coefficient and mean of the pitch means in every voiced region; Energy - maximum, mean and variance; RMS energy - maximum and mean - and Loudness. The features related with the voice quality and shared by all the categories are less than the prosodic and they specially refer to the third formant mean, the first and second formants bandwidth and the level of the activation of the speech signal; in this case, the maximum and mean stand out among all the voiced regions. These common features in all groups could be considered as the more relevant in order to design a system that intends to achieve full speaker independence. This system should be able to classify automatically emotions no matter who the speaker is.

The non-shared features in each subgroup should be analyzed in order to establish the relationships between these features and language and gender dependent characteristics.

## Conclusions and Future Work

This paper shows an attempt to select the most significant features for emotion recognition in spoken Basque and Spanish Languages. RekEmozio database was used as experimental data set. Several ML paradigms were used for the emotion classification task. Experiments were executed in three different phases, using different sets of features as classification variables in each phase. Moreover, feature subset selection was applied at each phase in order to seek for the most relevant feature subset. The three phases approach has proven to be useful in order to check which ML paradigms provide the best results in emotion automatic recognition and provide initial results with different sets of features.

Results show an encouraging improvement in the accuracies obtained. From an initial emotion classification performance of about 48% for the initial set of 32 features, performance has increased up to 80% when EDA-FSS is applied to the whole set of features for the case of Basque language. For the Spanish language, although a bit smaller, the performance has also shown a noticeable increase from 41% up to almost 75%. It is worth noting that achieved results are approaching the emotion recognition rate obtained by humans when validating RekEmozio database.

Therefore, emotion recognition rates have been improved using the features defined in this paper, but it must also be taken into account that such improvement has been achieved after applying EDA for FSS. Concerning the classifiers used, accuracies have clearly improved over the results obtained using the full set of features. IB appears as the best classifier in most experiments, if EDA-FSS is applied, and ID3 when no FSS is applied. In order to check the validity of achieved results, a greedy FSS searching approach (FSS-Forward) has been applied, but providing disappointing classification performances, and showing the best results when NB classifier is used. As future work, the authors will extend the study to other classifiers (SVM,…) and other methods of feature selection.

Authors have developed affective recognizers for speech using the categorical theory of emotions. However, currently they are studying emotions according to dimensional and appraisal models, information from other modalities (such as verbal and psycho physiological information) and also, other models such as user context models. In the future, the authors will perform studies related with the meaning of the utterances, comparing the results with semantically meaningful content and with non-semantically meaningful content. Moreover, more languages will be taken into account (such as the Catalan language).

## Supporting Information

File S1 **Feature matrix corresponding to Basque male actor M1.**
(CSV)

File S2 **Feature matrix corresponding to Basque male actor M2.**
(CSV)

File S3 **Feature matrix corresponding to Basque male actor M3.**
(CSV)

File S4 **Feature matrix corresponding to Basque male actor M4.**
(CSV)

File S5 **Feature matrix corresponding to Basque female actress F1.**
(CSV)

File S6 **Feature matrix corresponding to Basque female actress F2.**
(CSV)

File S7 **Feature matrix corresponding to Basque female actress F3.**
(CSV)

File S8 **Feature matrix corresponding to Spanish male actor M1.**
(CSV)

File S9 **Feature matrix corresponding to Spanish male actor M2.**
(CSV)

File S10 **Feature matrix corresponding to Spanish male actor M3.**
(CSV)

File S11 **Feature matrix corresponding to Spanish male actor M4.**
(CSV)

File S12 **Feature matrix corresponding to Spanish male actor M5.**
(CSV)

File S13 **Feature matrix corresponding to Spanish female actress F1.**
(CSV)

File 14 **Feature matrix corresponding to Spanish female actress F2.**
(CSV)

File S15 **Feature matrix corresponding to Spanish female actress F3.**
(CSV)

File S16 **Feature matrix corresponding to Spanish female actress F4.**
(CSV)

File S17 **Feature matrix corresponding to Spanish female actress F5.**
(CSV)
